# Comparison of prostatic urethral lift (PUL) versus water vapour thermal therapy (WVTT): A focus on drug‐free status and unplanned admissions

**DOI:** 10.1002/bco2.70242

**Published:** 2026-07-03

**Authors:** Athena Y. H. Lee, Brian W. H. Siu, Kelly H. Y. Tsang, Chi Ho Leung, Jeremy M. H. Ho, Yvonne Y. Y. Chan, Jeremy Y. C. Teoh, Peter K. F. Chiu, Ka Lun Lo, Chi Fai Ng

**Affiliations:** ^1^ S.H. Ho Urology Centre, Department of Surgery, Faculty of Medicine The Chinese University of Hong Kong Hong Kong China

**Keywords:** 6‐month drug‐free status, benign prostatic hyperplasia (BPH), minimally invasive surgical therapies (MIST), multivariable logistic regression, propensity score matching, prostatic urethral lift (PUL), unplanned readmission, water vapour thermal therapy (WVTT)

## Abstract

**Objectives:**

To compare prostatic urethral lift (PUL) and water vapour thermal therapy (WVTT) for benign prostatic hyperplasia (BPH) in a real‐world setting, this study compares clinical management outcomes that are less thoroughly documented in current literature—such as drug‐free status, complications and post‐procedure readmission rates.

**Patients and methods:**

BPH patients (age ≥50) with prostate volumes 30–80 cc who underwent either PUL or WVTT between January 2021 and December 2024 were identified. Patients were given BPH medications (alpha‐blockers and/or 5‐alpha reductase inhibitors) till 3 months postoperatively. The two groups were matched using a 1:3 propensity score matching; multivariable logistic regression analyses were performed on the full unmatched cohort. The primary outcome is drug‐free status, defined as the cessation of alpha‐blockers and 5‐alpha reductase inhibitors at 6 months post‐procedure. Secondary outcomes include unplanned readmission within 30 days.

**Results:**

In total, 421 eligible patients were analysed (363 WVTT; 58 PUL). In propensity score matched analyses (174 WVTT; 58 PUL), patients were more likely to be drug‐free after PUL at 6 months compared to WVTT (weighted OR: 2.62 [95% CI: 1.22–5.61], *p* = 0.013). The association remained significant in multivariable regression analysis (adjusted OR: 2.41 [95% CI: 1.31–4.58], *p* = 0.006). No statistically significant difference in 30‐day readmission rates was found between the two groups in either the propensity score (*p* = 0.097) or multivariable analysis (*p* = 0.123).

**Conclusion:**

In this retrospective propensity‐score‐matched study, PUL was associated with less BPH medication use at 6 months; readmission rates were comparable for PUL and WVTT.

## 1 INTRODUCTION

Benign prostatic hyperplasia (BPH) is a common condition affecting ageing men. Globally, more than 112.5 million patients are suffering from BPH in 2021.^1^ In Hong Kong, BPH is the most common underlying cause of lower urinary tract symptoms (LUTS),^2^ with 69.3% of men experiencing moderate‐to‐severe symptoms on the International Prostate Symptom Score (IPSS).^3^ Complications related to BPH include acute and chronic urinary retention, haematuria resulting from prostate bleed, urinary tract infection, bladder stones, bladder wall damage, renal dysfunction, incontinence and erectile dysfunction.^4^ BPH is also associated with problems such as sleep disturbance, decreased social activity and impaired sexual life,^5^ significantly impairing patients' quality of life.

While transurethral resection of the prostate (TURP) remains the gold standard for surgical management,^6^ it necessitates general or neuraxial anaesthesia while also carrying risks of erectile dysfunction and incontinence.^7^ As alternatives, minimally invasive surgical therapies (MIST) such as prostatic urethral lift (PUL) (UroLift™) and water vapour thermal therapy (WVTT) (Rezūm™) have gained popularity in recent years.^8^ WVTT is a minimally invasive therapy for benign prostatic enlargement which uses the thermal energy of a water stream for treatment.^9^, ^10^, ^11^ Water vapour is delivered to the prostate through a retractable vapour needle and disperses quickly through the tissue to disrupt cell membranes. The large amounts of thermal energy are from a central source at a higher temperature to a lower one; convection is used to heat tissue within the treatment zone to achieve prostate size reduction.^12^ Its counterpart, PUL, is a non‐ablative technique that utilises the deployment of adjustable implants to retract obstructing prostate adenoma to create a patent voiding channel through the prostatic fossa.^13^, ^14^

Studies have demonstrated that WVTT and PUL both show durable improvement in IPSS for 5 years without a clinically significant impact on sexual function post‐treatment.^15^ A UK comparative review has shown that the probability of experiencing ejaculation dysfunction drops from 66.75% for TURP to only 0‐3% for PUL and WVTT.^7^ Apart from treating LUTS, recent studies have demonstrated the safety and efficacy of WVTT and PUL in treating catheter‐dependent refractory retention.^16^, ^17^ Notably, a literature review by Leong et al. reported reduced hospitalisation, shorter operation time and reduced need for long‐term catheterisation after MIST, potentially minimising overall treatment costs and relieving the financial burden of the healthcare sector.^18^ This study does not, however, provide a head‐to‐head comparison between WVTT and PUL.

Due to their favourable safety profiles and preservation of sexual function, MIST has emerged as a treatment modality that appears to be desirable for both patients and healthcare systems alike, especially for prostates under 80 cc according to current guidelines.^19^ One of the latest publications, a large‐scale multicentre analysis (*n* = 6640), provided a head‐to‐head comparison between the two novel treatment methods. It revealed that patients who underwent WVTT had higher rates of postoperative haematuria, dysuria, urinary retention, fever, erectile dysfunction, urinary tract infections and emergency department visits compared to the PUL group.^20^ The same study also showed the reintervention rate for WVTT was lower than the PUL group at the first year, third year and fifth year across all age groups. The results align with previous literature, which also showed lower reintervention rates and higher complication rates in WVTT than in PUL cohorts.^21^ However, since this study is a secondary analysis of existing data from 144 organisations worldwide, differing inclusion criteria across centres and their selection bias could impact the accuracy of the analysis. Additionally, details of surgical procedures that might influence surgical outcomes were not clearly documented, such as the number of implants used in PUL or the number of ablations performed in WVTT.^20^ There is still no consensus on the favourability of these two procedures, which complicates physicians' decision‐making in developing treatment plans.

In light of current literature, several key questions about the outcomes of WVTT and PUL remain unanswered. For instance, the proportion of patients who achieve drug‐free status—an indicator of satisfactory postoperative voiding function—after both MIST procedures is unknown, since most trials focus on symptom scores rather than medication use cessation. Drug‐free status is clinically significant because it not only helps patients avoid medication side effects and improves their quality of life but also enhances cost‐effectiveness within healthcare systems.^22^ Another limitation of existing research is the uncertainty about whether real‐world unplanned readmission rates are higher due to variations in patient selection and postoperative management. Further research is required to identify predictors of reintervention, such as preoperative catheter dependence, baseline comorbidities and prostate size, which are currently not well characterised in trial data. Therefore, our study aims to perform a retrospective analysis of prospectively collected data on patients receiving either WVTT or PUL for BPH at our centre, to compare drug‐free status at 6 months post‐procedure, unplanned treatment‐related hospital admissions within 30 days and reintervention rates at 1 year for these two procedures.

## 2 METHODOLOGY

### 2.1 Data source and ethical approval

This retrospective study of a prospective cohort received ethical approval from our institutional ethics committee (CRE2022.049) prior to patient recruitment. The registry was established at three hospitals managed by a single urological team in Hong Kong, China. Written informed consent was obtained from all participants, and the study was conducted in accordance with the Declaration of Helsinki.

In our unit, all patients scheduled for surgical treatment of BPH, including TURP or MIST, undergo a standardised preoperative assessment and postoperative follow‐up at 3 and 12 months. All preoperative information, including patient data, premorbid conditions, indication for surgery, IPSS, uroflowmetry, prostate size, etc., as well as operation records, readmission records, and follow‐up details, is prospectively entered into our database, which forms the basis for this study.

The Hong Kong Hospital Authority (HA) is the primary public healthcare provider for the region's population of 7.4 million, managing approximately 94% of secondary and tertiary care. Additional follow‐up information in this study, such as drug records and unplanned hospital admissions, was retrieved from our Clinical Management System (CMS) and the Clinical Data Analysis and Reporting System (CDARS) of the HA. CDARS is a comprehensive database containing anonymized patient demographics, diagnoses (coded using ICD‐9), procedures, laboratory results, prescriptions and healthcare utilization records. ICD‐9 coding is performed by clinicians and dedicated administrative staff independent of the research team, with internal audits ensuring data integrity. The validity of CDARS for territory‐wide observational studies is well established, with published studies demonstrating a coding accuracy of 85%–99%.^23^

This would help to provide comprehensive admission and medication records for patients, even if they received treatment or medication from other hospitals in Hong Kong.

### 2.2 Study design and patient selection

The study population comprised male patients aged 50 years or older who underwent either the WVTT or PUL procedure for BPH between January 2021 and December 2024. After the urologist's assessment for eligibility and obtaining informed consent, patients underwent a standard preoperative assessment as mentioned before, including demographic data collection, uroflowmetry and prostate size measurement via transrectal ultrasonography (by the prolate ellipsoid formula). Eligible participants were required to have documented prostate volumes between 30 and 80 cc, as confirmed by transrectal ultrasound (TRUS) or magnetic resonance imaging (MRI). Both catheter‐dependent patients and non‐catheter‐dependent individuals were included to reflect real‐world clinical practice.

Patients were excluded if they had a prior history of prostate surgery (excluding diagnostic biopsies) or concurrent diagnoses of prostate cancer or neurogenic bladder dysfunction.

BPH medications [alpha‐blockers and/or 5‐alpha reductase inhibitors (5ARIs)] were prescribed till 3 months post‐op per protocol, thus drug‐free status was assessed at 6 months instead of 3 months.

### 2.3 Surgical procedure

Both WVTT and PUL were performed as ambulatory procedures under local anaesthesia. Preoperative urine culture was mandatory and asymptomatic bacteriuria was treated with a 1‐week course of antibiotics. Intravenous antibiotics were given within 1 h prior to the procedure, either according to previous culture or our local microbiogram.

WVTT was performed using the Rezūm system (Boston Scientific Company Inc., Marlborough, MA, USA) as previously described in literature.^24^ Periprostatic block with 1% lignocaine was injected under transrectal ultrasonography guidance. Patients were discharged with an indwelling catheter. In general, the first trial without catheter (TWOC) was on day 7 post‐op for non‐catheter‐dependent patients and day 14 post‐op for catheter‐dependent patients. In case of failed TWOC, patients were allowed another trial 2 weeks later and so on until 3 months. The rationale was that minimal prostate shrinkage was observed after 3 months in previous studies.^12^

PUL was performed using the PUL® System (Neotract Inc., Teleflex, Pleasanton, CA). Local anaesthesia was modified from previously published protocols^25^ using cold (4°C) 2% lignocaine gel injected via the urethra before cystoscope insertion. Post‐procedure, an indwelling catheter was not routinely placed unless the patient had a post‐void residual volume (PVR) exceeding 200 ml. Patients were discharged on the same day if they could void spontaneously with a PVR less than or equal to 200 ml. For patients discharged with a catheter, a subsequent trial without a catheter (TWOC) was scheduled 2 weeks after the operation, and if the patient failed TWOC again, another TWOC was scheduled 2 weeks later, repeating this process up to 3 months—in line with the protocol for WVTT.

### 2.4 Outcome measures and follow‐up protocol

Patient follow‐up began on the date of the initial surgical procedure (index date). It continued until the occurrence of a predefined study endpoint, death or the final follow‐up date of June 2025, whichever occurred first. The primary study outcome focused on drug‐free status, which was operationally defined as the complete cessation of alpha‐blockers and 5ARIs at both 6 months following the procedure. Secondary outcomes of interest encompassed several clinically relevant measures. These included unplanned hospital admissions occurring within 30 days post‐procedure, particularly those related to procedure‐associated complications such as urinary retention that necessitated catheterization, urinary tract infections or significant haematuria. The study also examined reintervention rates within the first postoperative year.

### 2.5 Statistical approach

To address potential selection bias between patients receiving WVTT and PUL treatments, the analysis employed a propensity score‐matched design. The propensity scores were generated through logistic regression modelling that incorporated multiple baseline characteristics, including patient age, body mass index, prostate size, catheter dependence status, diabetes, ischaemic heart disease and cerebrovascular disease. The matching process utilized a 1:3 optimal matching without replacement to optimize group balance. Matching was performed using the ‘MatchIt’ package in R.^26^ Assessment of covariate balance between matched groups was performed using standardized mean differences, with values below 0.1 considered indicative of satisfactory balance. Logistic regression models were employed to evaluate the treatment effect on drug‐free status and 30‐day readmission rate. The average treatment effect in the treated was estimated. Robust variances were used to calculate the 95% confidence intervals. To complement the primary matched analysis and assess the robustness of our findings, we conducted conventional multivariable logistic regression analyses on the full unmatched cohort. These models adjusted for the same covariates used in the propensity score model, providing an alternative approach to confounding control. All statistical testing employed two‐tailed approaches with a predetermined significance threshold of *p* < 0.05, and all analyses were performed using R software (version 4.3.1).

### 2.6 Subgroup analysis

Pre‐specified subgroup analyses were conducted to examine potential treatment effect heterogeneity across clinically relevant patient subgroups, including catheter dependence status and involvement of median lobe in treatments.

## 3 RESULTS

In total, 421 patients were eligible for this study and included in the analysis, comprising 363 patients in the WVTT group and 58 in the PUL group. After propensity score matching, 174 patients remained in the WVTT group, while all 58 patients in the PUL group were retained. Baseline characteristics of the patients before and after matching are summarized in Table 1a. The two groups were well‐balanced post‐matching, with absolute standardized differences (ASD) for all parameters below 0.1.

**TABLE 1a:** Baseline clinical characteristics and balancing diagnostics before and after propensity score matching.

Parameters	Before PS matching				After PS matching			
	WVTT (*n* = 363)	PUL (*n* = 58)	P	ASD	WVTT (*n* = 174)	PUL (*n* = 58)	P	ASD
Age, years	73.0 ± 8.5	72.0 ± 7.8	0.374	0.13	72.6 ± 8.6	72.0 ± 7.8	0.614	0.08
BMI, kg/m^2^	23.5 ± 3.4	24.1 ± 4.3	0.318	0.14	24.0 ± 3.6	24.1 ± 4.3	0.875	0.02
Prostate size, cc	54.5 ± 15.2	51.5 ± 12.7	0.106	0.24	52.0 ± 15.4	51.5 ± 12.7	0.826	0.04
Catheter‐dependent	257 (70.8)	34 (58.6)	0.062	0.25	105 (60.3)	34 (58.6)	0.818	0.04
DM	57 (15.7)	14 (24.1)	0.111	0.20	42 (24.1)	14 (24.1)	>0.9	0
IHD	34 (9.4)	12 (20.7)	0.010	0.28	30 (17.2)	12 (20.7)	0.570	0.09
CVA	39 (10.7)	11 (19.0)	0.072	0.21	30 (17.2)	11 (19.0)	0.771	0.04

*Note*: Continuous variables were presented as mean ± standard deviation and compared by *t*‐test, while categorical variables were presented as number (percentage) and compared by chi‐square test.

Abbreviations: ASD, absolute standardized difference; BMI, body mass index; CVA, cerebrovascular accident; DM, diabetes mellitus; IHD, ischaemia heart disease.

In the matched cohort, intraoperative and short‐term postoperative outcomes were compared between the WVTT and PUL groups, with continuous variables presented as median (interquartile range [IQR]) (Table 1b). Regarding intraoperative parameters, the median operative time was comparable between the two groups: 20 min (IQR 15–25) in the WVTT group and 20 min (IQR 16–25) in the PUL group. The time to trial without catheter (TWOC) was substantially longer in the WVTT group (median 12 days, IQR 18–29 days) compared to the PUL group, where the median TWOC was 0 days (IQR 0–0 days).

**TABLE 1b:** Intra‐operative and post‐operative outcomes of the matched cohort.

	WVTT (*n* = 174)	PUL (*n* = 58)
Operative time	20 (15–25)	20 (16–25)
Number of cycles/tabs	8 (6–11)	4 (4–5)
Percentage with median lobe	55 (31.6%)	11 (19.0%)
Time to TWOC	12 (18–29)	0 (0–0)
30‐day readmission rate	30 (17.2%)	5 (8.6%)
3‐month IPSS	5 (3–8)	8 (4–12)
3‐month QoL	2 (2–3)	3 (2–3)
3‐month Qmax	15.5 (11.7–20.1)	12.6 (9.8–16.0)
6‐month drug‐free rate	76 (43.7%)	38 (65.5%)

*Note*: Continuous variables were presented as median (interquartile range), while categorical variables were presented as number (percentage).

Abbreviations: IPSS, International Prostate Symptom Score; Qmax, maximum flow rate; QoL, Quality of Life; TWOC, trial without catheter.

At the 3‐month postoperative follow‐up, functional outcomes were evaluated using the IPSS, quality of life (QoL) score and maximum urinary flow rate (Qmax). The WVTT group had a median IPSS of 5 (IQR 3–8), median QoL score of 2 (IQR 2–3) and median Qmax of 15.5 ml/s (IQR 11.7–20.1). On the other hand, the PUL group had a median IPSS of 8 (IQR 4–12), a median IPSS‐QoL score of 3 (IQR 2–3) and a median Qmax of 12.6 ml/s (IQR 9.8–16.0 ml/s).

### 3.1 Drug‐free status, 30‐day readmission rate and reintervention rate

The results of multivariable regression and propensity score matching for drug‐free status and readmission rates were summarised in Table 2a. In matched analyses, more men in the PUL cohort achieved drug‐free status at 6 months compared to WVTT (weighted OR: 2.62 [95% CI: 1.22–5.61], *p* = 0.013). The association remained significant in multivariable regression analysis (adjusted OR: 2.41 [95% CI: 1.31–4.58], *p* = 0.006).

**TABLE 2a:** Summary of results of multivariable analyses and propensity score matching (WVTT as reference) on drug‐free status and readmission rate.

Multivariable regression^a^	OR (95% CI)	*p*
6‐month drug‐free status	2.41 (1.31–4.58)	0.006
30‐day readmission rate	0.46 (0.15–1.13)	0.123

Propensity score matching	OR (95% CI)	*p*
6‐month drug‐free status	2.62 (1.22–5.61)	0.013
30‐day readmission rate	0.45 (0.17–1.16)	0.097

Abbreviations: CI, confidence interval; OR, odds ratio.

^a^ Adjusted for age, catheter dependence, BMI, prostate volume, diabetes mellitus, ischaemic heart disease and cerebrovascular accident.

No statistically significant difference in 30‐day readmission rates was found between the two groups in either the propensity score (*p* = 0.097) or multivariable analysis (*p* = 0.123). This lack of significance may be due to the low number of readmission events (WVTT: 30; PUL: 5). The reasons for admission in the matched cohort are summarized in Table 2b. Consequently, caution is warranted in interpreting any perceived readmission benefits until larger studies can validate these findings.

**TABLE 2b:** Causes of readmission in the matched cohort.

Causes of readmission	WVTT (*n* = 30)	PUL (*n* = 5)
Haematuria	11 (36.7%)	1 (20%)
Urinary tract infection	7 (23.3%)	0 (0%)
Recurrent retention	6 (20%)	1 (20%)
Sepsis	1 (3.3%)	0 (0%)
Other causes	5 (16.7%)	3 (60%)

At 12 months, within the matched cohort (174 WVTT; 58 PUL), 97.1% of patients in the WVTT group and 100% in the PUL group remained catheter‐free.

### 3.2 Subgroup analysis

The subgroup analysis in Table 3 examined the 6‐month drug‐free status across different patient demographics, consistently using WVTT as the reference group. Drug‐free status favoured PUL in catheter‐dependent patients (OR 2.54, 95% CI 1.13–5.70, *p* = 0.024) and notably in patients without median lobe treatment (OR 2.53, 95% CI 1.18–5.43, *p* = 0.018).

**TABLE 3:** Subgroup analysis on 6‐month drug‐free status (WVTT as reference).

	No. of patients	OR (95% CI)	*p*	*p for interaction*
Catheter‐dependent				0.891
Yes	139	2.54 (1.13–5.70)	0.024	
No	93	2.31 (0.74–7.27)	0.151	
Median lobe treated				0.792
Yes	66	2.10 (0.59–7.50)	0.253	
No	161	2.53 (1.18–5.43)	0.018	

Abbreviations: CI, confidence interval; OR, odds ratio.

## 4 DISCUSSION

This retrospective analysis of a prospective cohort of patients with BPH undergoing MISTs showed that men undergoing PUL were more likely to be drug‐free than their counterparts at 6 months after treatment.

Postoperative discontinuation of BPH medication is an under‐reported outcome in studies. Although it is not a direct measure of symptoms or the degree of obstruction [such as IPSS, Qmax or bladder outlet obstruction index (BOOI)], it holds significant relevance, especially in real‐world settings. Discontinuing alpha‐blockers or 5ARIs can help avoid potential adverse effects associated with the drugs, especially those related to sexual function. It also has implications for cost‐effectiveness within the healthcare system,^27^ and might be an additional motivating factor for both the patient and healthcare administrators to consider the shift of medical therapy to MIST for BPH.

While the traditional treatment algorithm for LUTS always begins with medications, the concept of MIST as a first‐line interventional therapy (FIT) is emerging, where MIST serves as a bridge between medication and surgery. In theory, an ideal FIT should enable rapid and safe recovery, facilitate discontinuation of medication and delay or avoid high‐risk surgical interventions.^28^ Therefore, as MIST continues to gain popularity, rates of BPH medication discontinuation and unplanned readmissions should be of high importance in clinical studies.

Our study demonstrated men undergoing PUL were more likely to be drug‐free at 6 months after treatment. WVTT and PUL create a prostatic channel effectively via different mechanism: The mechanical nature of PUL, which lifts and holds the enlarged prostate tissue away from the urethra without ablation, may contribute to this early symptomatic relief. Although it has been shown on MRI that the gadolinium‐enhancing ablative lesions of WVTT do not cross zonal boundaries and do not persist past 6 months,^12^ it is plausible that the residual effects of the 103‐degree water vapour may cause irritative symptoms, leading to less medication discontinuation. A 5‐year analysis of post‐surgical medication use among patients treated with PUL, TURP and photoselective vapourization of prostate (PVP) revealed that the post‐treatment medication usage rates were similar across all three procedures.^29^ It challenges the assumption that TURP and PVP universally eliminate the need for medical therapy, making MIST a comparable alternative to traditional BPH surgeries.

Catheter‐dependent patients undergoing PUL were more likely to be drug‐free at 6 months after treatment. The PULSAR prospective controlled trial (a prospective controlled trial of 51 catheter‐dependent men) demonstrated that 73% of catheter‐dependent men were catheter‐ and surgery‐free at 12 months post‐PUL, with key predictors of success being a shorter pre‐procedural catheter duration, lower baseline PSA and younger patient age^16^; the efficacy of PUL in catheter‐dependent patients has also been demonstrated in real‐world settings.^30^, ^31^ While WVTT also showed promise in our local cohort,^17^ with over 90% catheter‐free rate and a median catheter time of 14 days, the results in the current study could aid urologists in counselling catheter‐dependent patients prior to considering MIST.

Furthermore, the absence of a median lobe is associated with superior drug‐free outcomes following PUL when compared to WVTT in this study. The L.I.F.T. trial, which established PUL's efficacy, specifically excluded men with obstructive median lobes (OML). While the MedLift study suggested OML can be effectively and safely treated by PUL,^32^ those with OML had a higher catheterization rate, attributed to the learning curve and increased manipulation. If the OML is not adequately retracted into the prostatic fossa, not only does it result in suboptimal symptom improvement, but there is also risk of implanting into the bladder. Cases of bladder stone formation secondary to displaced/misplaced tabs requiring reoperation for transurethral removal have been reported.^33^

While short of statistical significance, the 30‐day readmission rate of WVTT doubled that of PUL. A key contributing factor to readmissions following WVTT is the presence of an indwelling catheter. Indwelling catheters are associated with both infectious and non‐infectious complications.^34^ After PUL, an indwelling catheter was not routinely placed unless the patient had a PVR exceeding 200 ml; however, all patients were discharged with indwelling catheters for WVTT. The CLEAR trial, a head‐to‐head randomized controlled study comparing WVTT and PUL, provided additional insights into early postoperative recovery. PUL patients demonstrated significantly better catheter independence, sexual function and overall satisfaction within the first month post‐procedure (NCT04338776). Most side effects of PUL are transient, including haematuria, dysuria, urgency, pelvic discomfort and urge incontinence.^35^

However, despite these early advantages, long‐term safety and durability data favour WVTT.^20^ A pivotal study by McVary et al. using the Accordion Severity Grading System reported that WVTT was associated with a significantly lower 5‐year postoperative morbidity index compared to PUL, indicating fewer long‐term complications and adverse events.^36^ Additionally, WVTT has consistently shown lower retreatment rates over time, reinforcing its role as a durable option for patients seeking sustained symptom relief.^20^ These findings emphasise the importance of individualised treatment selection, balancing short‐term recovery benefits with long‐term durability and complication profiles.

Several limitations of the study should be acknowledged. First, patients were given BPH medications till 3 months postoperatively per protocol; thus, 6‐month drug‐free status was chosen as the primary outcome. Consequently, its timepoint does not coincide with the timing of IPSS/Qmax measurement at 3 months. Second, there could be confounding factors explaining the restarting of BPH medications, for example, family physicians prescribing alpha‐blockers for blood pressure control and patients resuming BPH medications due to personal preference instead of suboptimal symptom control. Third, as a retrospective analysis of a prospective registry, this study inherently carries a risk of bias due to the lack of blinding and randomization.

Nonetheless, propensity score matching was performed to limit confounding—with age, prostate volume, catheter dependence and key comorbidities matched between PUL and WVTT. The robustness of the findings was supported by sensitivity analyses using both propensity score matching and multivariable regression of the unmatched model to account for residual confoundings. Importantly, our study focused on important and under‐reported real‐world outcomes—unplanned readmission rates and medication discontinuation rates. Prospective and randomized trials are warranted before definitive conclusions can be made on the comparison between WVTT and PUL.

## 5 CONCLUSION

In this retrospective propensity score‐matched study, PUL was associated with less BPH medication use at 6 months; readmission rates were comparable for PUL and WVTT. Potential confounding may arise as there were no strict clinical criteria guiding the resumption of BPH medications; prospective randomized trials are warranted to confirm the findings. Both procedures demonstrated sustained voiding improvement with no reinterventions required within 1 year.

## AUTHOR CONTRIBUTIONS

**Athena Y. H. Lee:** Conceptualization; data curation; formal analysis; methodology; writing—original draft preparation; writing—review and editing. **Brian W. H. Siu:** Conceptualization; data curation; formal analysis; methodology; writing—original draft preparation; writing—review and editing; project administration; investigation. **Kelly H. Y. Tsang:** Data curation; formal analysis; writing—review and editing. **Chi Ho Leung:** Formal analysis; writing—review and editing. **Jeremy M. H. Ho:** Data curation; writing—review and editing. **Yvonne Y. Y. Chan:** Data curation; writing—review and editing. **Jeremy Y. C. Teoh:** Supervision; writing—review and editing. **Peter K. F. Chiu:** Conceptualization; methodology; supervision; writing—review and editing. **Ka Lun Lo:** Conceptualization; methodology; supervision; writing—review and editing. **Chi Fai Ng:** Conceptualization; methodology; project administration; resources; supervision; writing—review and editing.

## CONFLICT OF INTEREST STATEMENT

The authors declare that no conflicts exist.
